# Relationship between heart rate variability and pulse wave velocity and their association with patient outcomes in chronic kidney disease 

**DOI:** 10.5414/CN108020

**Published:** 2013-12-20

**Authors:** Preeti Chandra, Robin L. Sands, Brenda W. Gillespie, Nathan W. Levin, Peter Kotanko, Margaret Kiser, Fredric Finkelstein, Alan Hinderliter, Sanjay Rajagopalan, David Sengstock, Rajiv Saran

**Affiliations:** 1 Department of Internal Medicine, University of Maryland, Baltimore, MD,; 2 University of Michigan-Kidney Epidemiology and Cost Center, Ann Arbor, MI,; 3 Renal Research Institute, New York, NY,; 4 Department of Internal Medicine, University of North Carolina, Chapel Hill, NC,; 5 Hospital of St. Raphael Yale University, New Haven, CT,; 6 Department of Internal Medicine, University of North Carolina, Chapel Hill, NC,; 7 Department of Cardiovascular Medicine, Ohio State University, Columbus, OH,; 8 Oakwood Healthcare System, Dearborn, MI, and; 9 Department of Internal Medicine, University of Michigan, Ann Arbor, MI, USA

**Keywords:** autonomic nervous system, cardiovascular disease risk factors, cardiovascular outcomes, cohort study, end stage renal disease

## Abstract

Background: Arterial stiffness and low heart rate variability (HRV) have each been associated with increased cardiovascular risk in a variety of patient populations. We explored the relationship between HRV and pulse wave velocity (PWV measure of arterial stiffness) in patients with chronic kidney disease (CKD prior to ESRD) along with examining their association with the outcomes of cardiovascular disease (CVD), death, and progression to end stage renal disease (ESRD). Methods: The RRI-CKD Study is a 4-center prospective cohort study of CKD stages 3 – 5 (n = 834). A subset underwent both HRV testing by 24-hour Holter and carotid-femoral PWV (n = 240). Multiple linear regression was used to assess predictors of PWV and Cox regression to investigate the association of HRV and PWV with time to first CVD event or death and ESRD. Results: Although several HRV measures were inversely correlated with PWV, this association was attenuated after adjustment for age and/or diabetes and no longer significant after adjustment for C-reactive protein. Low HRV and high PWV were individually associated with increased risk of the composite endpoint of CVD/death in multivariable analysis. The risk of the composite of CVD/death was highest for patients with both low HRV and high PWV. Conclusion: Age, diabetes, and inflammation together explained the inverse association between HRV and PWV. Inflammation may play a role in the pathogenesis of both low HRV and high PWV. The combination of low HRV and high PWV showed the strongest association with a composite CVD outcome. Mechanisms underlying abnormalities in PWV and HRV, and the role of these measures as intermediate outcomes in future trials in CKD patients, merit further study.

## Introduction 

Arterial stiffness as measured by pulse wave velocity (PWV) is associated with increased risk of cardiovascular disease (CVD) and overall mortality both in patients with hypertension and those with end stage renal disease (ESRD) on dialysis [1, 2, 3, 4]. Several factors such as higher blood pressure (BP), dyslipidemia, diabetes mellitus, inflammation, and calcification, among others could underlie the pathogenesis of a higher PWV in chronic kidney disease (CKD) [4, 5]. Higher PWV has also been linked with lower kidney function in many though not all studies [6, 7, 8]. CKD is associated with altered autonomic regulation with increased sympathetic activity [9]. Autonomic dysfunction as measured by low heart rate variability (HRV) is associated with increased cardiovascular and overall mortality in diabetics, CVD, and patients with kidney disease [10, 11]. Autonomic dysfunction has been shown to be inversely related to PWV in patients with diabetes [12, 13, 14, 15] and also in patients on dialysis [16], but there is only limited data on the non-dialysis CKD population [17]. 

We postulated that autonomic dysfunction (increased sympathetic activity, in particular) in those with CKD would be associated with increased arterial stiffness. We additionally sought to examine the combined risk posed by alterations in HRV and PWV in predicting the composite of CVD and/or death and ESRD outcomes in a CKD cohort. 

## Methods 

The Renal Research Institute (RRI)-CKD Study is a 4-center, prospective cohort study of adults with moderate-to-severe CKD (stages 3 – 5) enrolled between June 2000 and February 2006 (n = 834). The study methodology has been published previously [18]. Briefly, eligibility criteria included age ≥ 18 years and estimated glomerular filtration rate (eGFR) ≤ 50 mL/min by the Cockroft-Gault formula. Subsequently, the abbreviated (4-variable) Modification of Diet in Renal Disease (MDRD) equation was used and eGFR was between 50 and 60 mL/min/1.73m^2^ in 14 subjects. To exclude patients with transient renal impairment prior to enrollment, GFR was estimated on two occasions at least 1 month apart. At enrollment and follow-up visits, data on demographic characteristics, anthropometric measures, cause of CKD, symptoms, laboratory values, and medication data were collected. From January 1, 2003 onwards, individuals from the original RRI-CKD cohort (n = 627) were invited to undergo non-invasive cardiovascular studies, including pulse wave velocity and 24-hour Holter monitoring as part of a cardiovascular (CV) sub-study. 

**Figure 1.**
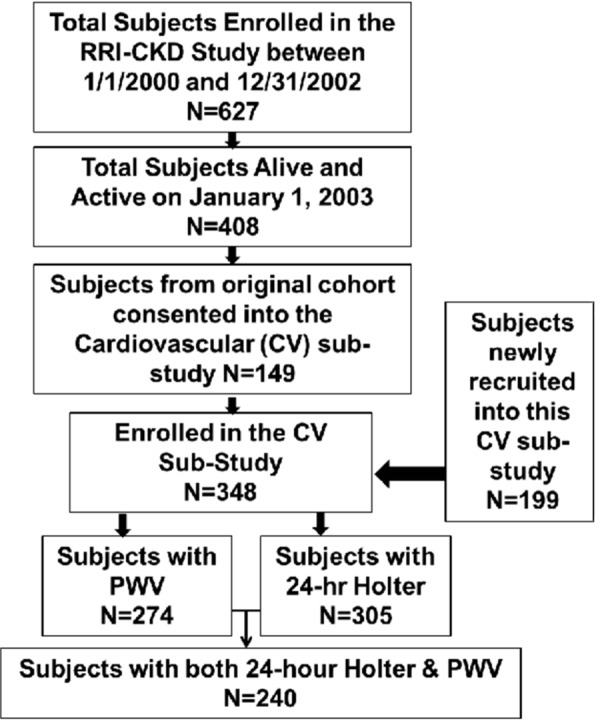
Patient recruitment flow into the cardiovascular sub-study of the RRI-CKD study (n = 305).

Figure 1 displays patient recruitment flow into this CV sub-study. Patients who consented into the new CV sub-study from the original RRI-CKD study (n = 149) were generally healthier than those who did not; they were younger (mean age 58 vs. 64), had higher mean eGFR (27 vs. 25), and fewer had diabetes (30% vs. 42%) or history of CVD (37% vs. 58%). However, the 199 newly recruited patients into the CVD sub-study – while similar to these original RRI-CKD cohort participants with respect to age, diabetes, hypertension, history of CVD, race, gender, and medication use – had significantly higher mean eGFR (32 vs. 24). The difference between the CV sub-study and original RRI-CKD participants was due to patient self-selection into a study that involved substantial testing and patient burden. This selection seems especially likely because the characteristics of the newly recruited CV sub-study patients were similar to the consenters from the original RRI-CKD cohort. Finally, of the 348 patients who entered into the CV sub-study, 43 declined Holter monitoring. These patients were otherwise similar to the 305 who underwent this procedure, except for significantly older mean age (67 vs. 60). There were no significant differences between patients who underwent PWV measurement and and those who declined (n = 74). The present analysis includes the 240 patients who underwent both HRV and PWV measurements. 

Study coordinators at each site were trained in Holter monitoring and other study procedures at the University of Michigan Data Coordinating Center (DCC) to ensure uniformity of technique across centers. During the 24-hour Holter, patients went about their usual activities but were asked to refrain from heavy physical activity. The Holter data were transferred electronically via a file transfer protocol (FTP) to the DCC where they were analyzed using SyneTec Holter analysis software, version 1.20 (Ela Medical, Paris, France) under the supervision of the study cardiologist (SR). The 24-hour tracing was examined, and masking was used to exclude areas of artifact. Abnormal atrial and ventricular rhythms were manually verified. Time and frequency domain measures were calculated for the 24-hour period, day (8:00 AM – 9:00 PM) and night periods. Patients on nitroglycerin patch, with defibrillator, active pacing, or with allergy to electrode adhesive material were excluded. The terminology/abbreviations pertaining to the HRV parameters collected in this study are shown in Table S1. 

“Central” arterial stiffness was assessed using carotid-femoral PWV. Systolic and diastolic blood pressures (SBP, DBP) were measured after a 5-minute rest in the supine position. The length of the descending aorta was approximated by subtracting the manubrium-carotid distance from the manubrium-femoral artery distance. A hand-held tonometer was placed over the carotid and then femoral arteries to record pressure waves. Simultaneous EKG tracings were recorded. PWV was calculated in an automated fashion (AtCor version 7.0). Four measurements were recorded for each subject. A measurement was excluded if the pressure contour was of poor quality or a > 15% difference in heart rate (HR) was found between the carotid and femoral measurements. A subject’s PWV was the average of the acceptable measurements. 

The outcomes studied were the composite of CVD/Death and ESRD. CVD events included those related to coronary artery disease (CAD), cerebrovascular disease, peripheral arterial disease, congestive heart failure (CHF), and cardiac arrest. ESRD was defined by documented initiation of dialysis or preemptive renal transplantation. All outcomes were ascertained on an ongoing basis by study coordinators through regular review of electronic health records, direct patient contact in clinic, and periodic telephone communication. CVD events to be collected were pre-specified and identified using a CVD event data collection form. Follow-up of patients ended on December 31, 2006. Study protocol was approved at all sites by their individual Institutional Review Boards. All subjects provided written informed consent. 

### Statistical Analysis 

Multivariable regression models were used to explore the association between HRV and PWV after simultaneous adjustment for variables associated with PWV in univariate analysis and/or factors known to affect PWV. The method of best subsets with the R-squared selection criterion guided the model selection process [19]. HRV parameters were then added one at a time to this multivariable linear regression model to assess the independent effect of HRV on PWV. However, since several of the clinical variables included in this model were also significantly correlated with HRV, the HRV-PWV association was further explored by examining regression models with increasing adjustment for the significant predictors of PWV in multivariable analyses. Cox regression was used to analyze time-to-event outcomes. All variables from Table 1 were included to determine the clinical predictors of the composite outcome of CVD/Death and ESRD, and the method of best subsets with the likelihood score (chi-square) statistic selection criterion was used to guide model selection. These models are referred to as “base” models for each outcome (Table S5). Each measure of HRV and PWV were then added to this “base” model to assess their independent associations with each outcome. Models with and without medications were tested, however, final models were selected without medications; this did not significantly affect the results. Skewed variables were natural log transformed and a p-value < 0.05 was considered significant. All analyses were conducted using SAS, version 9.2 (SAS Institute Inc., Cary, NC, USA). 

A previous publication yielded similar outcomes for using 24-hour, night and day HRV measures [11]; for the purpose of this manuscript, we only present 24-hour HRV measures for analysis Furthermore, only those 24-h HRV measures that were significantly associated with PWV (LF, LF/HF, VLF, TP, ASDNN) in univariate analyses were further explored in subsequent regression models and survival analysis. Pearson correlations among these selected 24-hour HRV measures in this CKD cohort are reported in Table S2. 

## Results 

Baseline patient characteristics at the time of non-invasive cardiovascular testing are displayed in Table 1. The average age was 60 ± 15 years, 53% were male, 78% white, and 18% black. A majority were hypertensive (89%), 30% were diabetic, 35% had a history of CVD, and 47% were smokers. Table 2 shows baseline measurements of HRV and PWV overall and by presence or absence of diabetes. These comparisons were made to validate the known link between diabetes and both autonomic dysfunction and increased vascular stiffness (i.e., low HRV and high PWV respectively) [8, 11]. In this CKD cohort, PWV was significantly higher and most HRV measures were significantly lower in diabetics than in non-diabetics. 

### Associations of PWV with baseline clinical characteristics and HRV 

**Figure 2.**
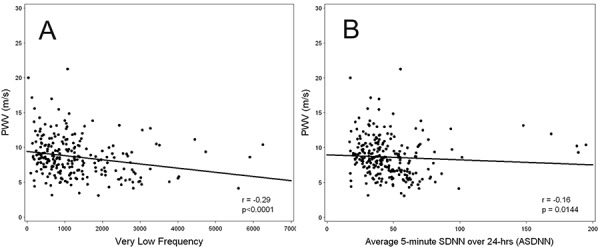
Scatter plot and least squares regression line for pulse wave velocity (PWV) versus (A) very low frequency and (B) average of 5-minute standard deviation of all normal to normal R-R intervals over 24 hours (ASDNN).

Table S3 displays univariate associations of patient characteristics with PWV. Several clinically important variables such as older age, diabetes (DM), hypertension, history of CVD, higher values of: systolic blood pressure (SBP), heart rate (HR; p = 0.056), body mass index (BMI), corrected serum calcium, fasting blood glucose, or C-reactive protein (CRP), and use of diuretics or calcium channel blockers were all significantly associated with higher PWV. No association was observed between total cholesterol, LDL, HDL or triglycerides, serum phosphorus, albumin, hemoglobin, eGFR, smoking, use of beta-blockers or ACE/ARB with PWV. Several time and frequency domain measures of HRV (LF, VLF, LF/HF ratio, total power (TP) and ASDNN) were inversely correlated with PWV and with low HRV associated with high PWV (Figure 2) (Table 4). 

In multivariable analyses, older age, higher SBP, higher HR, DM, higher BMI, and elevated CRP were significantly associated with high PWV (Table 3). After simultaneous adjustment for these variables, PWV was no longer significantly associated with HRV. However, since several of these variables were also associated with HRV in this cohort (Table 4), the association of HRV and PWV with increasing adjustment for these variables was also explored (Table S4). Model 1 displays the significant unadjusted inverse HRV-PWV association. Models 2a – 2d show a slight attenuation of this association after adjustment for age, DM, CRP, or HR. The addition of either SBP (Model 3b) or BMI (Model 3c), or both (Model 4b) to the age-adjusted model did not significantly alter the age-adjusted HRV-PWV association. Although the addition of diabetes did further attenuate the magnitude of the age-adjusted HRV-PWV association (Model 3a), ASDNN and TP remained significantly associated with PWV. However, the relationship between HRV and PWV is no longer significant with the addition of either HR (Model 3d) or CRP (Model 3e) to the age-adjusted model or age and diabetes adjusted model (Model 4d, e). 

### Association of PWV and HRV with clinical outcomes 

In this cohort, 37 patients had at least one CVD event, 9 patients died, and 53 reached ESRD. 

In unadjusted analyses, both high PWV and low HRV were associated with increased risk of CVD. In multivariable analyses, the risk of the composite outcome of first CVD event or death was higher in males, those with prior history of CVD and with low serum albumin. (Table 5). After adjusting for these variables, low HRV and high PWV were each significantly associated with higher risk of CVD/death (Table 5(A)). 

Increased risk of ESRD was associated with lower baseline eGFR, higher urine albumin to creatinine ratio, and higher iPTH in multivariable analysis (Table 5). PWV was not associated with risk of ESRD whereas amongst HRV measures, only VLF was significant (Table 5 (B)). 

**Figure 3.**
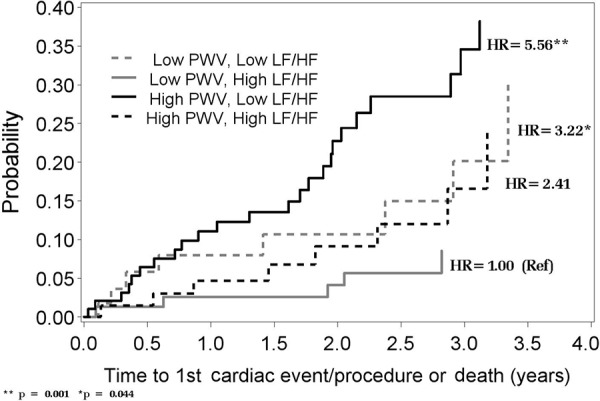
The cumulative probability of first cardiovascular disease (CVD) event or death over time since Holter monitoring by higher or lower (above/below median) PWV and HRV (median PWV = 8.35 m/s; median Ln(LF/HF) ratio = 1.26 ms^2^). Plotted values were calculated based on Cox regression adjusted for mean values of: age (60 years), phosphorus (3.7), albumin (4.0), proportion with history of CVD (0.35), and proportion male (0.53).

When both HRV and PWV are placed together in the model predicting CVD/death, both HRV and PWV are associated with increased risk of CVD/death (Table 5 (A)). As shown in Figure 3, patients with either a low LF/HF ratio and/or high PWV were associated with a higher risk of CVD/death compared to patients with both a high LF/HF ratio and low PWV. The risk of CVD/death was highest among patients with both a low LF/HF and a high PWV. Similar findings were observed with other HRV measures (LF, VLF, ASDNN, and total power-data not shown). 

## Discussion 

CKD has been linked with both increased sympathetic activity as well as higher arterial stiffness, both of which are associated with adverse cardiovascular outcomes and mortality in this population [2, 3, 4, 5, 6, 7, 9, 10, 11]. We undertook this analysis to (i) explore the relationship between autonomic dysfunction (as assessed by HRV) and arterial stiffness (as measured by PWV) in a CKD cohort and (ii) to assess the association of both HRV and PWV (individually and combined) on patient outcomes. 

We had postulated that we would find an inverse relationship between PWV and HRV among patients with CKD, consistent with prior experimental evidence outlined below. We found that HRV was indeed significantly inversely correlated with PWV in unadjusted analyses. However, after progressively adjusting for confounders, particularly CRP and HR, this association was no longer significant (Table S4). Heart rate is an important influence on PWV [20], and heart rate is intimately linked with HRV. Similarly, CRP has been associated with low HRV in this patient cohort [11]. CRP was also associated with higher PWV in this cohort, consistent with prior literature [21]. In addition, CRP was inversely related to heart rate in this cohort (R = 0.31, < 0.0001). Hence, heart rate and CRP are important confounders in the HRV-PWV relationship. In prior work, HRV has been associated with arterial stiffness in both type 1 and type 2 diabetics although these analyses were not adjusted for heart rate [12, 13, 14], while a study by Ittersum et al. [15] showed that aortic PWV was associated with mean autonomic score adjusted for age, BP, and heart rate. 

The relationship between autonomic nervous system and arterial stiffness, however, remains plausible. Increased sympathetic activity with resultant increased heart rate can cause damage to elastin fibers resulting in replacement with collagen and resultant decreased elasticity and compliance. In direct support of a causal relationship between activation of the sympathetic nervous system and abnormalities in fibrosis/loss of arterial compliance, sympathectomy in rats results in increased distensibility (and hence more compliance) in both muscular arteries, such as femoral as well as larger elastic arteries, such as carotid artery. This increase in distensibility was independent of decrease in blood pressure resulting from sympathectomy [22]. The same authors have also suggested that in the larger elastic carotid arteries in rats, reduced distensibility from pacing induced tachycardia is likely more related to the viscous properties of the vessel wall and inertial response to intravascular pressure than to the smooth muscle tone [23]. In a study by Sa Cunha et al. [24], high PWV was associated with higher heart rate and lower baroreceptor sensitivity after adjustment for age and systolic BP in patients with hypertension. Fatigue and fracture of the elastic fibers in the arterial wall are the major causes of increased arterial stiffness with aging; this alteration is more prominent in the larger “elastic” arteries that pulsate to a greater degree than the “muscular” arteries like the femoral [25]. There is experimental evidence that cyclic stretching stimulates production of increased matrix proteins by aortic smooth muscle cells in vitro [22], and that increased hemodynamic stress (product of heart rate and BP) is associated with increased aortoiliac atherosclerosis in experimental animals [26]. 

In this CKD cohort, higher PWV was associated with greater risk of the composite outcome of first CVD event or death in multivariable analysis. This is consistent with prior studies from different populations including ESRD [1, 2, 3, 4] and underscores the importance of arterial stiffness as an adverse prognostic indicator. HRV measures (LF, LF/HF, VLF, TP, and ASDNN) were also associated with CVD/death risk in this cohort as previously reported [11]. When both PWV and any of these HRV measures are placed in the same model predicting the risk of CVD/death, PWV and HRV are independently associated with these outcomes, with the highest risk observed among patients with both a low HRV and a high PWV. These findings suggest consideration of both arterial stiffness and HRV together provide a greater prognostic significance compared to consideration of either measure alone. Data from larger cohorts with both measurements would be desirable to further substantiate these findings. 

While one measure of HRV (namely VLF) was associated with increased risk for ESRD, an association between PWV and risk to ESRD was not observed in this cohort. A weak association between HRV and ESRD in a larger sample from this cohort was also reported in a previous publication [11]. It is not clear why PWV was not associated with ESRD in this CKD cohort. We could speculate that there is a relationship, but this study has failed to uncover it because of a relatively small sample size and/or short duration of follow-up. However, the latter possibility is less likely, given that there were a sizable number of ESRD events in this cohort; however, a previous publication discusses the lack of association between PWV and eGFR in this cohort [8]. 

This study has several strengths. The associations were studied in a fairly large number of CKD patients followed longitudinally, with near simultaneous baseline measurements of arterial stiffness and heart rate variability, allowing us to investigate the integrated influence of these parameters in the same analysis, along with their potential associations. To the best of our knowledge, there are no other studies in the CKD literature looking at these parameters simultaneously with respect to their combined association with outcomes. Furthermore, arterial stiffness was measured at the aorta by a standardized technique that has been validated in previous studies. HRV was measured by a 24-hour Holter, which remains the gold standard. Availability of demographic and important baseline clinical data allowed us to perform multivariable analyses. 

Certain limitations of this study need to be acknowledged. As with any observational study, we can only comment on associations and not causality. The outcomes, while systematically collected by study coordinators, were not adjudicated by committee. The number of deaths was small in this referred CKD clinic-based cohort; we therefore studied the composite outcome of CVD or death. Direct measurements of sympathetic activity were not part of the original study design. However, based on the published literature, heart rate variability is well-recognized as a surrogate of autonomic imbalance. 

## Summary and conclusion 

This study describes association between autonomic dysfunction as measured by HRV and arterial stiffness as measured by PWV in a multicenter prospective CKD cohort. HRV appears to be inversely correlated with PWV. However, this correlation was explained by age, presence of diabetes, and CRP levels, suggesting that underlying inflammation may well be an important confounder of this relationship and may mediate abnormalities in both parameters. Both low HRV and high PWV were associated with higher risk, the composite of CVD/death in this cohort, with the risk of CVD/death being highest in those patients with a combination of both a low HRV and a high PWV. Future research should further examine these associations in larger cohorts and clarify underlying mechanisms in patients with CKD with the aim of identifying populations at greatest risk for adverse CVD events. Both HRV and PWV could be considered for investigation as potential intermediate outcome measures in future clinical trials for this high risk patient population. 

## Acknowledgements 

The study was funded by the Renal Research Institute, New York, NY, USA. We are grateful to all study coordinators and Ms. Kerri Briesmiester, Project Manager, for training all site study coordinators in the Holter and Pulse Wave Velocity technique. 

The study was conducted with the support of the Michigan Clinical Research Unit, University of Michigan, funded by the NIH grant UL1RR024986. 

Preeti Chandra, MD was supported by a National Kidney Foundation Research Fellowship award from 2009 to 2011. These results have previously been presented in part at the American Society of Nephrology in 2010 as a poster presentation. 

Drs. Rajiv Saran, MD and Brenda W. Gillespie, PhD are members of the project “Establishing a Surveillance System for Chronic Kidney Disease” supported under a cooperative agreement from the Centers for Disease Control and Prevention through grant number 1U58DP003836. 

## Conflicts of interest 

Drs. Peter Kotanko and Nathan Levin hold stock in Fresenius. No other authors have any declared conflicts of interest. 

**Table 1.** Patients characteristics at the time of non-invasive cardiovascular testing in the RRI-chronic kidney disease (CKD) cohort (n = 240^a^).

Variable	Mean (SD) or median (min, max) or % (n)
Demographics/anthropometrics	
Age	60 (15)
Body mass index (kg/m^2^)	28.6 (5.9)
Gender: male	53% (128)
Race: white	78% (187)
Race: black	18% (43)
Race: other	4% (10)
Current/former smoker	47% (113)
Cardiovascular Indices	
Total cholesterol (mg/dL)	92 ± 54
Systolic blood pressure (mm/Hg)	139 ± 23
Diastolic blood pressure (mm/Hg)	78 ± 13
Comorbidities	
Diabetes	30% (72)
Hypertension	89% (214)
Cardiovascular disease	35% (85)
Cause of CKD^b^	
Diabetes	25% (59)
Hypertension	51% (122)
Other	58% (140)
Medications	
Diuretics	49% (118)
ACE inhibitors or A-II receptor blockers	70% (167)
Beta blocker	50% (119)
Calcium channel blocker	42% (101)
Erythropoiesis-stimulating agent	24% (57)
Statin	45% (108)
Laboratory values	
C-reactive protein (mg/L)	1.7 (0, 50)
Intact parathyroid hormone (ng/mL)	111 (5, 991)
Estimated GFR (mL/min/1.73m^2^)	29 (12)
Serum creatinine (mg/dL)	2.2 (1, 11)
Serum albumin (g/dL)	4.0 (0.5)
Blood urea nitrogen (mg/dL)	38 (14, 131)
Corrected serum calcium (mg/dL)	9.2 (0.6)
Serum glucose (mg/dL)	97 (46, 393)
Serum phosphorus (mg/dL)	3.7 (0.9)
Serum potassium (mEq/L)	4.5 (0.6)
Hemoglobin (g/dL)	12.1 (1.6)
Urine albumin/creatinine ratio	191 (2, 9259)

^a^n = 239 for BMI, albumin, and calcium; 238 ≤ n ≤ 234 for phosphorus, iPTH, BUN, cholesterol, and potassium; 230 ≤ n ≤ 228 for Alb/Cr ratio, glucose, and CRP; and n = 219 for HG. ^b^Multiple causes of CKD are possible.

**Table 2.** Pulse wave velocity (PWV) and heart rate variability (HRV) measurements at the time of non-invasive cardiovascular testing in the RRI-chronic kidney disease (CKD) cohort. Continuous variables are reported as mean (standard deviation) for normally distributed variables and as median (min, max) for skewed variables. Categorical variables are reported as % (n). Significant differences between those with and without diabetes are shown in **bold** (p < 0.05).

Measure	Overall (n = 240^a^)	Diabetics (n = 72^a^)	Non-diabetics (n = 168)
Pulse wave velocity (m/s)	8.6 (2.8)	**10.3 (3.2)**	**7.9 (2.3)**
HRV time domain (ms)			
Standard deviation (SD) of all normal to normal R-R (NN) intervals	105 (36,306)	**92 (36, 306)**	**108 (48, 227)**
SD of 5-min average NN intervals (SDANN)	89 (25,258)	**78 (25, 258)**	**93 (39, 199)**
Average 5-minute SDNN over 24 h (ASDNN)	41 (17,195)	**36 (17, 188)**	**44 (18, 195)**
Root mean square of the difference of successive RR intervals (RMSSD)	24 (6, 267)	27 (6, 267)	23 (8, 260)
HRV frequency domain (ms^2^)			
Total power	1,554 (256, 30828)	**1,131 (256, 25058)**	**1,687 (297, 30828)**
Very low frequency (VLF)	1,014 (36, 6252)	**690 (36, 5908)**	**1,139 (101, 6252)**
Low frequency (LF)	310 (13, 11977)	**177 (13, 6688)**	**349 (24, 11977)**
High frequency (HF)	120 (5, 15123)	97 (5, 11247)	128 (11, 15123)
Low/high frequency ratio (LF/HF)	2.5 (0.2, 14)	**1.8 (0.3, 9.0)**	**2.9 (0.2, 13.9)**

^a^HRV frequency domain measures, n = 239 overall, n = 71 for diabetics.

**Table 3.** Multivariable linear regression models predicting pulse wave velocity in the RRI-chronic kidney disease (CKD) cohort.

Model 1			Model 2		
	n = 239	R^2^ = 0.46		n = 228	R^2^ = 0.46
Variable	β	p-value	Variable	β	p-value
Age	0.07	< 0.0001	Age	0.07	< 0.0001
Diabetes	1.46	< 0.0001	Diabetes	1.70	< 0.0001
Systolic blood pressure^a^ (mm/Hg)	0.03	< 0.0001	Systolic blood pressure^a^ (mm/Hg)	0.03	< 0.0001
Heart rate (bpm)	0.03	0.0059	Heart rate (bpm)	0.03	0.0398
Body mass index (kg/m^2^)	0.06	0.0068	C-reactive protein (mg/L)	0.29	0.0394

^a^Substitution of mean arterial pressure for systolic blood pressure yielded similar results.

**Table 4.** Pearson correlations of selected 24-hour HRV measures (log-scale) with significant predictors of PWV (and with PWV) in this CKD cohort.

	Age	Diabetes	Systolic blood pressure	Heart rate	Body mass index	C-reactive protein	Pulse wave velocity
HRV measure (over 24 h)	(years)		(mm/Hg)	(bpm)	(kg/m^2^)	(mg/L)	(m/s)
Time domain (ms)							
SD of 5-min average NN intervals (SDANN)	r = –0.02	r = –0.19**	r = –0.05	r = –0.35***	r = –0.08	r = –0.24***	r = –0.14*
Average 5-minute SDNN (ASDNN)	r = –0.03	r = –0.14*	r = –0.06	r = –0.32***	r = –0.02	r = –0.23***	r = –0.16*
Frequency domain (ms^2^)							
Total power	r = –0.03	r = –0.19**	r = –0.06	r = –0.33***	r = –0.03	r = –0.24***	r = –0.18*
Very low frequency (VLF)	r = –0.15*	r = –0.28***	r = –0.04	r = –0.42***	r = –0.06	r = –0.23***	r = –0.29***
Low frequency (LF)	r = –0.12	r = –0.24***	r = –0.12	r = –0.23***	r = –0.01	r = –0.21**	r = –0.22***
Low/high frequency ratio LF/HF)	r = –0.32***	r = –0.27***	r = –0.18*	r = –0.11	r = –0.12	r = –0.12	r = 0.26***

*p < 0.05, **p < 0.005, ***p < 0.0005.

**Table 5.** Adjusted Cox regression models predicting time to (A) composite of first cardiovascular disease (CVD) events or death and (B) ESRD endpoints. Starting with a base model for each endpoint (Table S5), either pulse wave velocity (PWV) or measure of heart rate variability (HRV; log-scale) was separately added to the base model (I) and then simultaneously added to the base model (II). Significant associations (p < 0.05) are in** bold** and marginal associations (p < 0.10) are shaded.

	I. Base model^a,b^ + PWV or HRV		II. Base model^a,b^ + PWV + HRV			
	Hazard ratio for PWV/HRV	p-value	Hazard ratio for HRV	p-value for HRV	HR for PWV	p-value for PWV
(A) First CVD event or death						
PWV (m/s)	**1.19 (1.09,1.31)**	0.0002				
ASDNN (ln ms)	**0.39 (0.18,0.83)**	0.0145	**0.46 (0.22,0.97)**	0.0400	**1.17 (1.07,1.28)**	0.0006
Total power (ln ms^2^)	**0.60 (0.40,0.89)**	0.0103	**0.66 (0.45,0.97)**	0.0355	**1.17 (1.07,1.28)**	0.0008
VLF (ln ms^2^)	**0.57 (0.41,0.79)**	0.0009	**0.69 (0.49,0.97)**	0.0304	**1.14 (1.03,1.25)**	0.0089
Low frequency (ln ms^2^)	**0.60 (0.45,0.80)**	0.0005	**0.65 (0.49,0.86)**	0.0025	**1.16 (1.06,1.27)**	0.0010
LF/HF ratio	**0.50 (0.35,0.71)**	< 0.0001	**0.53 (0.37,0.77)**	0.0007	**1.15 (1.05,1.26)**	0.0019
(B) ESRD						
PWV (m/s)	1.01 (0.91,1.12)	0.7934				
ASDNN (ln ms)	0.64 (0.33,1.23)	0.1810	0.63 (0.32,1.26)	0.1915	1.00 (0.90,1.11)	0.9521
Total power (ln ms^2^)	0.77 (0.55,1.09)	0.1399	0.77 (0.54,1.10)	0.1473	0.99 (0.89,1.11)	0.8875
VLF (ln ms^2^)	**0.66 (0.47,0.93)**	0.0191	**0.62 (0.41,0.92)**	0.0168	0.96 (0.85,1.07)	0.4391
Low frequency (ln ms^2^)	0.84 (0.65,1.07)	0.1563	0.83 (0.65,1.08)	0.1646	1.00 (0.90,1.11)	0.9482
LF/HF ratio	0.93 (0.64,1.36)	0.7167	0.94 (0.63,1.41)	0.7704	1.01 (0.90,1.12)	0.8830

ASDNN = Average 5-minute SDNN over 24 hours; VLF = very low frequency; LF/HF ratio = low/high frequency ratio. ^a^First CVD event or death: adjusted for h/o CVD, gender, and serum albumin (n = 238, 46 events, R^2^ = 0.11). ^b^ESRD: adjusted for eGFR, ln(ipth), and ln (Alb/CR) (n = 226, 51 events, R^2^ = 0.37).

**Table S1.** Overview of heart rate variability parameters.

Frequency domain HRV measures		Unit	Description
VLF	Power in very low frequency range	ms^2^	Physiological correlate unclear; may reflect vasomotor function, renin angiotensin system, and/or parasympathetic influence
LF	Power in low frequency range	ms^2^	Reflects sympathetic or sympathetic parasympathetic influence.
LF/HF	Ratio of low to high frequency power	–	Reflects sympathovagal balance.
TP	Total power	ms^2^	Estimate of overall HRV.
Time domain HRV measures		Unit	Description
ASDNN	Average of all 5-minute SDNN **over 24**	ms	Estimate of short-term components of HRV (i.e., changes in heart rate due to cycles < 5 minutes).

References: [10] Heart Rate Variability. Standards of measurement, physiological interpretation and clinical use, Task Force of European Society of Cardiology and the North American society of pacing and electrophysiology. Eur Heart J. 1996; 17: 354-381. *Kleiger RE, Stein PK, Bigger JT.* Heart Rate Variability: Measurement and Clinical Utility. Ann Noninvasive Electrocariol. 2005; *10:* 88-101.

**Table S2.** Pearson correlations among selected 24-h HRV measures (log-scale) in this CKD cohort.

HRV measure	ASDNN (ln ms)	Total power (ln ms^2^)	VLF (ln ms^2^)	Low frequency (ln ms^2^)	LF/HF ratio (ms^2^)
ASDNN (ms)	–	0.96**	0.81**	0.92**	–0.03
Total power (ln ms^2^)	0.96**	–	0.83**	0.91**	0.04
VLF (ln ms^2^)	0.81**	0.83**	–	0.77**	0.25**
Low frequency (ln ms^2^)	0.92**	0.91**	0.77**	–	0.14*
LF/HF ratio (ms^2^)	–0.03	0.04	0.25**	0.14*	–

*p < 0.05, **p < 0.0005.

**Table S3.** Univariate associations of patient characteristics with pulse wave velocity in the RRI-chronic kidney disease (CKD) cohort (n = 240^a^).

Variable Demographics/anthropometrics	β	p-value
Age	** 0.09**	**< 0.0001**
Gender: male	–0.29	0.3752
Race: white	0.33	0.4019
Body mass index (kg/m^2^)	** 0.09**	** 0.0013**
Current/former smoker	–0.41	0.4362
Cardiovascular indices		
Systolic blood pressure (mm/Hg)	**0.05**	**< 0.0001**
Diastolic blood pressure (mm/Hg)	–0.003	0.8008
Heart rate (bpm)	0.03	0.0568
Comorbidities		
Diabetes	**2.45**	**< 0.0001**
Hypertension	**0.97**	** 0.0438**
Cardiovascular disease	**1.29**	** 0.0002**
Medications		
Diuretics	** 0.92**	**0.0041**
ACE Inhibitors or A-II receptor Blockers	–0.50	0.1471
Beta blocker	0.27	0.3999
Calcium channel blocker	** 0.91**	**0.0052**
Erythropoiesis-stimulating agent	–0.13	0.7329
Statin	–0.16	0.6156
Laboratory values		
C-Reactive protein (mg/L)	**0.46**	**0.0100**
Intact parathyroid hormone (ng/mL)	0.08	0.6748
Estimated GFR (mL/min/1.73m^2^)	–0.004	0.7591
Serum creatinine (mg/dL)	–0.58	0.1406
Serum albumin (g/dL)	–0.46	0.1853
Blood urea nitrogen (mg/dL)	0.22	0.5668
Corrected serum calcium (mg/dL)	** 0.60**	**0.0265**
Serum glucose (mg/dL)	** 2.29**	**0.0008**
Serum phosphorus (mg/dL)	0.002	0.9924
Serum potassium (meq/L)	–0.06	0.8203
Hemoglobin (g/dL)	–0.08	0.4433
Total cholesterol (mg/dL)	0.01	0.0993
Low density lipoprotein (mg/dL)	0.01	0.1462
High density lipoprotein (mg/dL)	–0.003	0.8035
Triglycerides (mg/dL)	0.19	0.5154
Urine albumin/creatinine ratio	0.02	0.8203

^a^n = 239 for BMI, albumin, and calcium; 238 ≤ n ≤ 234 for phosphorus, iPTH, BUN, cholesterol, and potassium; 230 ≤ n ≤ 228 for Alb/Cr ratio, glucose, and CRP; and n = 219 for HG.

**Table S4.** Models display the association between pulse wave velocity (PWV) and heart rate variability (HRV; log-scale) with increasing adjustment for significant predictors of PWV in multivariable analyses (age, heart rate (HR)), C-reactive protein (CRP), diabetes (DM), and body mass index (BMI). Model estimates (β) and p-values from each model are reported. Significant associations are in **bold **(p < 0.05).

Y = PWV	(1) Unadjusted		(2a) Age-Adjusted		(2b) DM-Adjusted		(2c) HR-Adjusted		(2d) CRP-Adjusted	
HRV Measure	b	p-value	b	p-value	b	p-value	b	p-value	b	p-value
ASDNN (ms)	**–1.20**	**0.0026**	**–0.81**	**0.0196**	**–0.91**	**0.0167**	**–1.07**	**0.0119**	–0.79	0.0629
Total power (ms^2^)	**–0.63**	**0.0024**	**–0.44**	**0.0142**	**–0.46**	**0.0210**	**–0.56**	**0.0108**	**–0.44**	**0.0487**
VLF (ms^2^)	**–0.81**	**0.0001**	**–0.46**	**0.0098**	**–0.61**	**0.0023**	**–0.58**	**0.0003**	**–0.68**	**0.0025**
Low frequency (ms^2^)	**–0.62**	**0.0001**	**–0.34**	**0.0131**	**–0.46**	**0.0026**	**–0.78**	**0.0005**	**–0.48**	**0.0043**
LF/HF ratio (ms^2^)	**–0.78**	**0.0003**	–0.23	0.2444	**–0.56**	**0.0088**	**–0.76**	**0.0005**	**–0.61**	**0.0048**
2-Variable models:	(3a) Age + DM		(3b) Age + SBP		(3c) Age + BMI		(3d) Age + HR		(3e) Age + CRP^a^	
	b	p-value	b	p-value	b	p-value	b	p-value	b	p-value
ASDNN (ms)	**–0.66**	**0.0470**	**–0.78**	**0.0146**	**–0.81**	**0.0177**	–0.53	0.1439	–0.53	0.1538
Total power (ms^2^)	**–0.34**	**0.0489**	**–0.41**	**0.0123**	**–0.45**	**0.0117**	–0.30	0.1083	–0.32	0.0880
VLF (ms^2^)	–0.33	0.0618	**–0.47**	**0.0048**	**–0.45**	**0.0102**	–0.31	0.1134	–0.32	0.0889
Low frequency (ms^2^)	–0.24	0.0702	**–0.29**	**0.0232**	**–0.36**	**0.0088**	–0.27	0.0603	–0.23	0.1104
LF/HF ratio (ms^2^)	–0.06	0.7705	–0.09	0.6198	–0.19	0.3322	–0.18	0.3694	–0.19	0.3421
3-Variable models:	(4a) Age + DM + SBP		(4b) Age + SBP + BMI		(4c) Age + BMI + DM		(4d) Age + HR + DM		(4e) Age + CRP + DM	
	b	p-value	b	p-value	b	p-value	b	p-value	b	p-value
ASDNN (ms)	**–0.65**	**0.0357**	**–0.79**	**0.0116**	**–0.66**	**0.0433**	–0.44	0.2055	–0.39	0.2631
Total power (ms^2^)	**–0.33**	**0.0401**	**–0.42**	**0.0090**	**–0.35**	**0.0428**	–0.23	0.2078	–0.23	0.2065
VLF (ms^2^)	**–0.35**	**0.0315**	**–0.46**	**0.0047**	–0.32	0.0613	–0.19	0.3149	–0.18	0.3188
Low frequency (ms^2^)	–0.21	0.0934	**–0.31**	**0.0142**	–0.26	0.0524	–0.18	0.1908	–0.13	0.3437
LF/HF ratio (ms^2^)	0.04	0.8262	–0.05	0.7883	–0.04	0.8454	–0.01	0.9455	–0.02	0.9365

ASDNN = Average 5-minute SDNN over 24 hours, VLF = very low frequency, LF/HF ratio = low/high frequency ratio.

**Table S5.** Multivariable Cox regression models predicting time to (A). Composite of first cardiovascular disease (CVD) event or death and (B) ESRD endpoints.

Base model	HR (95% CI)	p-value
(A) First CVD event or death (46 events)	n = 238, R^2^ = 0.09	
History of CVD	2.69 (1.50,4.84)	0.0009
Males	1.83 (0.99,3.38)	0.0542
Albumin (g/dL)	2.69 (1.50,4.84)	0.0009
(B) ESRD endpoint (47 events)	n = 226, R^2^ = 0.37	
Estimated GFR (mL/min/1.73m^2^)	0.85 (0.81,0.89)	<0.0001
Urine albumin/creatinine ratio^a^	1.33 (1.12,1.58)	0.0013
Intact parathyroid hormone (ng/mL)^a^	1.44 (1.03,2.02)	0.0344

^a^Natural log scale.
